# Comparing Dose Calculation Algorithms for Heterogeneous Media: Analytical Anisotropic Algorithm Versus Acuros XB (Dm/Dw) With Continuous CT Value Variation

**DOI:** 10.7759/cureus.46805

**Published:** 2023-10-10

**Authors:** Yuya Yanagi, Kazuki Kubo, Takaaki Ito, Kenji Nakamura, Makoto Hirata, Hiroshi Doi, Hajime Monzen

**Affiliations:** 1 Department of Medical Physics, Graduate School of Medical Sciences, Kindai University, Osakasayama, JPN; 2 Department of Radiology, Shiga University of Medical Science Hospital, Otsu, JPN; 3 Department of Radiation Oncology, Faculty of Medicine, Kindai University, Osakasayama, JPN

**Keywords:** analytical anisotropic algorithm (aaa), heterogeneous material, hounsfield unit (hu), ct value, acuros xb (axb)

## Abstract

Background: To compare the doses calculated by the analytical anisotropic algorithm (AAA) and two dose reporting modes of Acuros XB (AXB(D_m_) and AXB(D_w_)) with varied CT values on the Eclipse (Varian Medical Systems, Palo Alto, CA).

Materials and methods: Virtual phantoms with a central layer of heterogeneous material (thickness = 2 or 5 cm) were created with Eclipse. Using single or opposed fields, the field sizes were 5 x 5 cm^2^ or 10 x 10 cm^2^. The photon energies were 6 or 10 MV, and the source-to-target distance was 100 cm. The relative doses at the center of the heterogeneous material layer were evaluated with varied CT values, from -1000 to 3000 HU. Values were normalized with the dose at 0 HU (100%) for comparative analysis.

Results: The results obtained from continuous data for a single field, 6 MV, 5 x 5 cm^2^, and the heterogeneous material 5 cm, where the differences between algorithms were most pronounced, were as follows. In the low-density region (-1000 HU and -800 HU), the dose differences for AXB with reference to AAA were, respectively, -54.5% and +4.6% (AXB(D_m_)) and -47.0% and +3.5% (AXB(D_w_)), and in the high-density regions (1000 HU and 3000 HU) were -5.7% and -8.8% (AXB(D_m_)) and +7.4% and +3.5% (AXB(D_w_)), respectively. Consequently, dose differences at arbitrary CT values could be obtained.

Conclusion: Dose differences between these algorithms were clarified for heterogeneous materials. The risk of dose reduction or escalation in clinical use was clearly visible between CT values from -1000 to 3000 HU.

## Introduction

The human body consists of a variety of tissues and cavities with different physical and radiological properties [1]. According to the American Association of Physicists in Medicine Task Group 65 (AAPM TG-65), the dose for these heterogeneous tissues should be calculated to an accuracy of 2-3%. To meet this requirement, dose calculation algorithms have evolved to include recoil electrons for heterogeneity correction [1]. The treatment planning system (TPS) Eclipse (Varian Medical Systems, Palo Alto, CA) is equipped with two main dose calculation algorithms: analytical anisotropic algorithm (AAA) and Acuros XB (AXB). AAA, which reports dose as the "dose to water-in-water (D_w,w_)," is a three-dimensional pencil beam convolution/superposition algorithm that handles the heterogeneity of anisotropic tissues by using a multi-directional photon scattering kernel based on water of varying densities [2-4]. AXB is based on linear Boltzmann transport equation (LBTE) solvers that allow for accurate modeling of dose deposition in media [2,5,6]. The two dose reporting modes of AXB, dose to medium (D_m_) and dose to water (D_w_), use the same transport calculation but different post-processing methods [2]. According to studies evaluating dose calculations for heterogeneous materials with different densities, AXB(D_m_) provides dose distributions close to those of measurements or Monte Carlo calculations [5-12].

Some studies have evaluated these algorithms in terms of the accuracy of dose calculations for six substances, i.e., air, lung, adipose, muscle, cartilage, and bone, as defined in the International Commission on Radiation Units & Measurements (ICRU) Report 23 [5,6,13]. For example, in the lung region (CT value of -990 to -377 HU), Fogliata et al. evaluated the dose for normal (0.198 g/cm^3^, -780 HU) and light lungs (0.035 g/cm^3^, -942 HU), and reported a discrepancy between AAA and AXB dose calculations for light lungs [5]. In cancerous lungs and even healthy lungs, the CT value varies widely, with an average change of 200 HU between maximal inspiration and expiration [14]. For this reason, evaluation of only specific densities or CT values is insufficient to evaluate dose calculations for lungs of various densities. Therefore, the data presenting the dose difference between the two dose calculation algorithms for continuously changing CT values must be useful in clinical practice. For other organs, there are no reported studies evaluating the variation in calculated doses between algorithms with continuous changes in the CT value or mass density, and thus the performance of these algorithms for these organs is unknown. Although such large dose differences might occur depending on the CT value, previous studies have only evaluated the dose discretely and have not evaluated the dose according to the detailed CT value. We hypothesized that the dose calculated using different algorithms may be different for the same organ with minor changes in CT values. Since it is unclear, we considered it necessary to examine it in detail. It may also be possible to easily estimate how much the dose difference between AAA and AXB will be by simply obtaining CT values, even in the region where large dose differences are produced.

In this study, we compared and evaluated the doses calculated by AAA, AXB(D_m_), and AXB(D_w_) for heterogeneous materials, with the CT value continuously varying from -1000 to 3000 HU in detail. The pitfalls of these algorithms for clinical use were discussed.

## Materials and methods

Equipment

**Figure 1:**
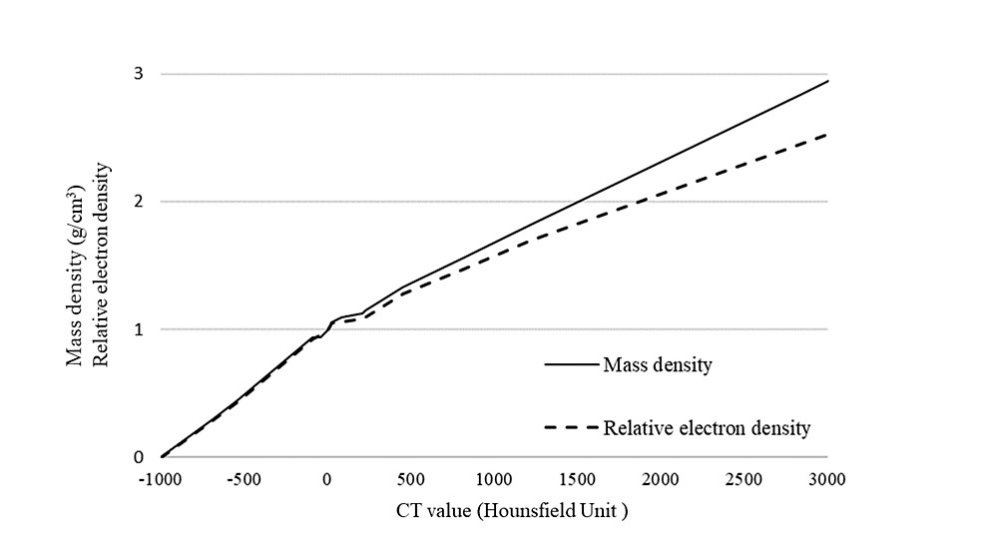
CT calibration curves obtained from measurements acquired at our institution. Air: -1000 to -786 HU; lung: -990 to -377 HU; adipose tissue: -441 to 4 HU; muscle/skeletal: -54 to 83 HU; cartilage: 30 to 874 HU; bone: 106 to 3000 HU.

To evaluate the AAA and AXB (D_m_ and D_w_) algorithms, dose calculations with virtual phantoms were performed on Eclipse TPS (version 13.6.23). The default values of the physical material table (version 13.5) in the photon and electron algorithms reference guide provided by Varian Medical Systems were used for AXB material assignments [2]. CT calibration curves for the conversion of CT values to mass density and relative electron density were measured at our institution (Figure 1). The Gammex model 467 tissue characterization phantom (GAMMEX, Middleton, MA) was used to obtain the table. CT scans were acquired using a 16-slice CT scanner LightSpeed RT 16 (GE HealthCare, Chicago, IL) at 120 kVp and 300 mAs with a slice thickness and slice interval of 2.5 mm and a field of view of 500 mm.

Relative dose evaluation using one layer of heterogeneous material in water-equivalent material: one-layer heterogeneous material phantom

**Figure 2:**
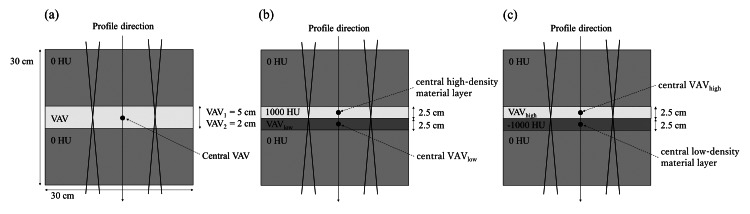
Virtual phantoms with variable assignment volume (VAV). Depth relative dose (RD) profiles were obtained along the beam center axis. (A) Heterogeneous material one-layer phantom (-1000 to 3000 HU) with VAV1 of 5 cm or VAV2 of 2 cm. (B) Heterogeneous material two-layer phantom. High-density material layer (1000 HU) and VAV_low_ (-1000 to 1000 HU) next to each other. (C) Heterogeneous material two-layer phantom. VAV_high_ (-1000 to 1000 HU) and low-density material layer (-1000 HU) next to each other.

The virtual phantom (30 × 30 × 30 cm^3^) assigned to 0 HU was created on the TPS, as shown in Figure 2A. One layer of variable assignment volume (VAV) was delineated in the virtual phantom (VAV1 of 5 cm thickness and VAV2 of 2 cm thickness). The CT value of each VAV varied from -1000 to 3000 HU on the TPS, every 10 HU in the range of -1000 to -900 HU, every 100 HU in the range of -900 to 1000 HU, every 20 HU in the range of -100 to 100 HU, and every 500 HU in the range of 1000 to 3000 HU. Based on the pre-study results, regions of CT values with greater dose variation and regions of CT values that are used more frequently in clinical practice were measured in more detail. Dose calculations were performed using the following parameters: photon energies of 6 and 10 MV, source-to-target distance (STD) of 100 cm, single fields at 0° or two opposed fields at 0° and 180°, 150 monitor units irradiated/fraction, and field size of 5 cm × 5 cm or 10 cm × 10 cm. The reason for irradiating the beam from both sides was that we considered the scenario in which beam attenuation was compensated by the opposed beam. The calculation grid size was 0.25 cm. The depth relative dose (RD) profiles were obtained from the dose profile along the beam center axis. The center of the VAV from the RD profile of each CT value was set as the evaluation point. The dose at 0 HU was normalized to 100%, and the relationship between the CT value and the RD was evaluated and compared for each dose calculation algorithm. The doses of each calculation algorithm at CT value 0 HU were almost equal (<±0.6%), confirming that they were correctly commissioned.

Relative dose evaluation using heterogeneous materials next to each other: two-layer heterogeneous material phantom

Because the one-layer heterogeneous material phantom above had a material of only 0 HU next to the VAV, each algorithm was used to calculate the dose for the case of two layers of different heterogeneous materials next to each other. Figure 2B shows the same virtual phantom setup with a 2.5 cm high-density material layer fixed at 1000 HU and a 2.5 cm VAV_low_ with variable CT values placed next to each other in the center. Similarly, Figure 2C shows a setup with a 2.5 cm low-density material layer fixed at -1000 HU and a 2.5 cm VAV_high_ with variable CT values placed next to each other. The CT values of VAV_low_ and VAV_high_ were varied from -1000 to 1000 HU on the TPS, every 50 HU in the range of -1000 to -900 HU, every 100 HU in the range of -900 to 300 HU, and after 300 HU, 500 HU, 800 HU, and 1000 HU. The dose calculation was performed as above, and the irradiation field size was 10 cm × 10 cm. In the same way, depth RD profiles were obtained. The center of each of the high-density material layers, low-density material layers, VAV_low_, and VAV_high_ was set as the evaluation point. The dose at 0 HU was normalized to 100%, and the relationship between the CT value and the RD was evaluated for each dose calculation algorithm.

## Results

Dose evaluation using one layer of heterogeneous material in water-equivalent material

**Figure 3:**
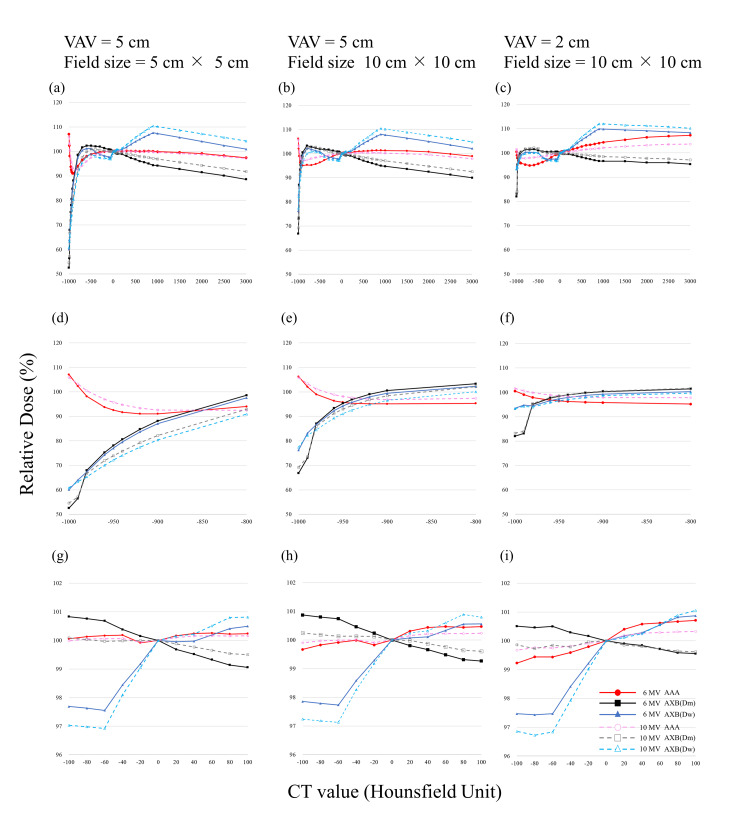
Relative dose for single fields at the center of the variable assignment volume (VAV) as a function of the CT value calculated by each algorithm, normalized with the dose for CT value of 0 HU set to 100%. (A) VAV of 5 cm and radiation field size (FS) of 5 cm x 5 cm. (B) VAV of 5 cm and FS of 10 cm x 10 cm. (C) VAV of 2 cm and FS of 10 cm x 10 cm. D-F are enlarged views, from -1000 to -800 HU, of A-C, respectively. G-I are enlarged views, from -100 to 100 HU, of A-C, respectively. AAA: analytical anisotropic algorithm; AXB: Acuros XB.

**Figure 4:**
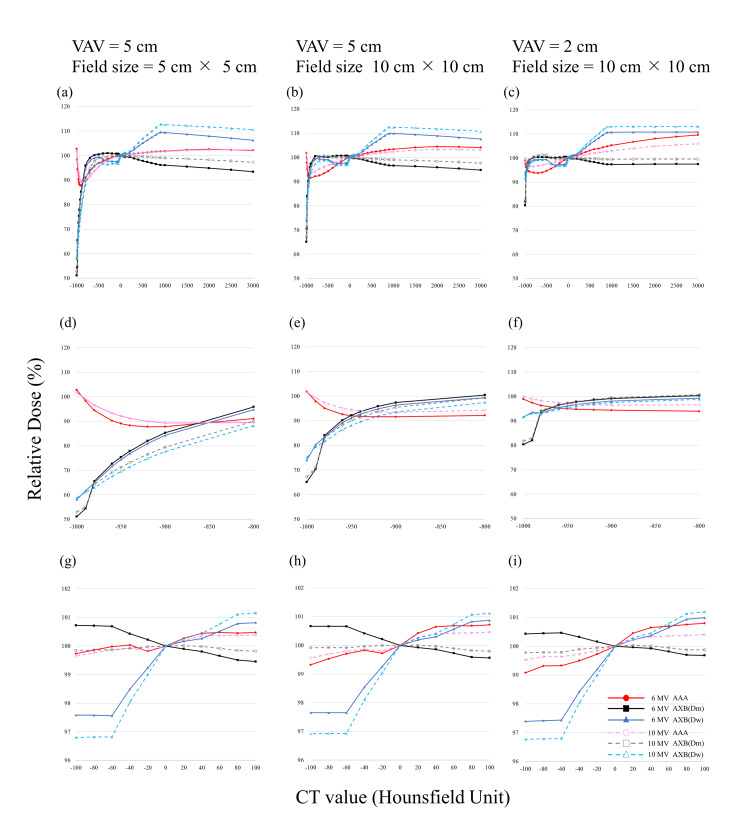
Relative dose for two opposed fields at the center of the variable assignment volume (VAV) as a function of the CT value calculated by each algorithm, normalized with the dose for CT value of 0 HU set to 100%. (A) VAV of 5 cm and radiation field size (FS) of 5 cm x 5 cm. (B) VAV of 5 cm and FS of 10 cm x 10 cm. (C) VAV of 2 cm and FS of 10 cm x 10 cm. D-F are enlarged views, from -1000 to -800 HU, of A-C, respectively. G-I are enlarged views, from -100 to 100 HU, of A-C, respectively. AAA: analytical anisotropic algorithm; AXB: Acuros XB.

In the present study, the RD for single and two opposed fields showed similar trends. The results of the RD for single fields and two opposed fields are shown in Figures 3, 4. The dose at 0 HU at the center of the VAV was normalized to 100%. In low-density regions, the dose difference between algorithms AAA and AXB was more than 50% higher for AAA in cases with a VAV path length of 5 cm and irradiation field size of 5 cm × 5 cm. The dose difference was larger for longer path lengths through the heterogeneous region and for smaller irradiation fields. In high-density regions, the dose difference was approximately 10% higher for AXB(D_w_) and 10% lower for AXB(D_m_) compared to AAA. The dose difference was not as large as in the low-density region. With a photon energy of 6 MV, VAV path length of 5 cm, and irradiation field size of 5 cm × 5 cm, the differences in the RD for single fields calculated by AXB with respect to that calculated by AAA in the low-density regions of -1000 HU and -800 HU were, respectively, -54.5% and +4.6% (AXB(D_m_)) and -47.0% and +3.5% (AXB(D_w_)). In contrast, the differences in the RD calculated by AXB with respect to that calculated by AAA in the high-density regions of 1000 HU and 3000 HU were, respectively, -5.7% and -8.8% (AXB(D_m_)) and +7.4% and +3.5% (AXB(D_w_)). The RD difference for each condition was maximal at -1000 HU, with maximum values between AAA and AXB(D_m_) at 6 MV, VAV of 5 cm, and irradiated field size of 5 cm × 5 cm (Figure 3A). In the low-density region (CT value ranging from -1000 to -800 HU), relatively large changes were observed (Figures 3D-3F). In the high-density region, the RD increased in the order AXB(D_m_) < AAA < AXB(D_w_). The results for two opposed fields showed generally similar trends to those with single fields, except that the dose was higher in the high-density region. The RDs calculated by each algorithm and obtained from linear interpolation for CT values of -1000 HU (air), -727 HU (lung), -108 HU (adipose), 26 HU (muscle), 106 HU (cartilage), and 1264 HU (bone) are shown in Table 1. The CT values were derived from the default densities provided by Varian Medical Systems [2]. The doses shown in the table were examples and the RDs at any CT value could be evaluated.

**Table 1:** Relative doses calculated by each calculation algorithm for CT values for each condition. Relative doses calculated by each calculation algorithm for CT values of -1000 HU (air), -727 HU (lung), -108 HU (adipose), 26 HU (muscle), 106 HU (cartilage), and 1264 HU (bone) for each condition, normalized with the dose for CT value of 0 HU set to 100%. VAV: variable assignment volume; AAA: analytical anisotropic algorithm; AXB: Acuros XB.

				Air	Lung	Adipose	Muscle	Cartilage	Bone
				-1000 HU	-727 HU	-108 HU	26 HU	106 HU	1264 HU
Single fields	6 MV	VAV 5 cm, FS 5 cm × 5 cm	AAA	107.1%	95.8%	100.0%	100.2%	100.2%	99.7%
			AXB(D_m_)	52.6%	100.8%	100.9%	99.6%	99.0%	93.5%
			AXB(D_w_)	60.1%	99.7%	97.7%	100.0%	100.5%	106.5%
		VAV 5 cm, FS 10 cm × 10 cm	AAA	106.2%	95.3%	99.6%	100.3%	100.5%	101.2%
			AXB(D_m_)	66.8%	103.0%	100.9%	99.8%	99.3%	94.2%
			AXB(D_w_)	76.3%	101.9%	97.9%	100.1%	100.6%	107.1%
		VAV 2 cm, FS 10 cm × 10 cm	AAA	100.5%	94.9%	99.1%	100.5%	100.7%	104.9%
			AXB(D_m_)	82.0%	101.3%	100.5%	99.9%	99.6%	96.6%
			AXB(D_w_)	93.5%	100.3%	97.5%	100.2%	100.9%	109.6%
	10 MV	VAV 5 cm, FS 5 cm × 5 cm	AAA	105.9%	93.7%	100.0%	100.1%	100.2%	99.4%
			AXB(D_m_)	54.6%	96.2%	100.1%	99.8%	99.5%	96.2%
			AXB(D_w_)	60.8%	94.3%	97.1%	100.2%	100.8%	109.4%
		VAV 5 cm, FS 10 cm × 10 cm	AAA	105.9%	97.8%	99.9%	100.2%	100.2%	100.1%
			AXB(D_m_)	69.2%	102.6%	100.3%	100.0%	99.6%	96.4%
			AXB(D_w_)	77.4%	100.6%	97.3%	100.3%	100.8%	109.5%
		VAV 2 cm, FS 10 cm × 10 cm	AAA	101.6%	97.9%	99.6%	100.2%	100.3%	102.4%
			AXB(D_m_)	83.2%	101.9%	99.9%	99.8%	99.6%	98.4%
			AXB(D_w_)	93.3%	99.9%	96.9%	100.2%	101.1%	111.7%
Opposed fields	6 MV	VAV 5 cm, FS 5 cm × 5 cm	AAA	102.8%	93.1%	99.7%	100.3%	100.5%	102.1%
			AXB(D_m_)	51.1%	98.2%	100.7%	99.9%	99.4%	95.7%
			AXB(D_w_)	58.1%	97.1%	97.6%	100.2%	100.8%	109.0%
		VAV 5 cm, FS 10 cm × 10 cm	AAA	101.8%	92.4%	99.2%	100.5%	100.7%	103.8%
			AXB(D_m_)	65.1%	100.4%	100.7%	99.9%	99.6%	96.4%
			AXB(D_w_)	74.0%	99.3%	97.7%	100.2%	100.9%	109.6%
		VAV 2 cm, FS 10 cm × 10 cm	AAA	98.9%	93.8%	99.0%	100.5%	100.8%	106.0%
			AXB(D_m_)	80.3%	100.4%	100.4%	100.0%	99.7%	97.4%
			AXB(D_w_)	91.6%	99.3%	97.4%	100.3%	101.0%	110.7%
	10 MV	VAV 5 cm, FS 5 cm × 5 cm	AAA	101.7%	91.0%	99.6%	100.2%	100.4%	101.9%
			AXB(D_m_)	53.0%	93.6%	99.8%	100.0%	99.8%	98.9%
			AXB(D_w_)	58.8%	91.7%	96.8%	100.3%	101.2%	112.3%
		VAV 5 cm, FS 10 cm × 10 cm	AAA	101.7%	95.0%	99.5%	100.3%	100.5%	102.6%
			AXB(D_m_)	67.2%	100.1%	99.9%	100.0%	99.8%	98.9%
			AXB(D_w_)	75.0%	98.2%	96.9%	100.3%	101.1%	112.3%
		VAV 2 cm, FS 10 cm × 10 cm	AAA	99.9%	96.7%	99.5%	100.3%	100.4%	103.5%
			AXB(D_m_)	81.8%	101.0%	99.8%	100.0%	99.9%	99.5%
			AXB(D_w_)	91.7%	99.1%	96.8%	100.3%	101.2%	113.0%

Dose evaluation using heterogeneous materials placed next to each other

**Figure 5:**
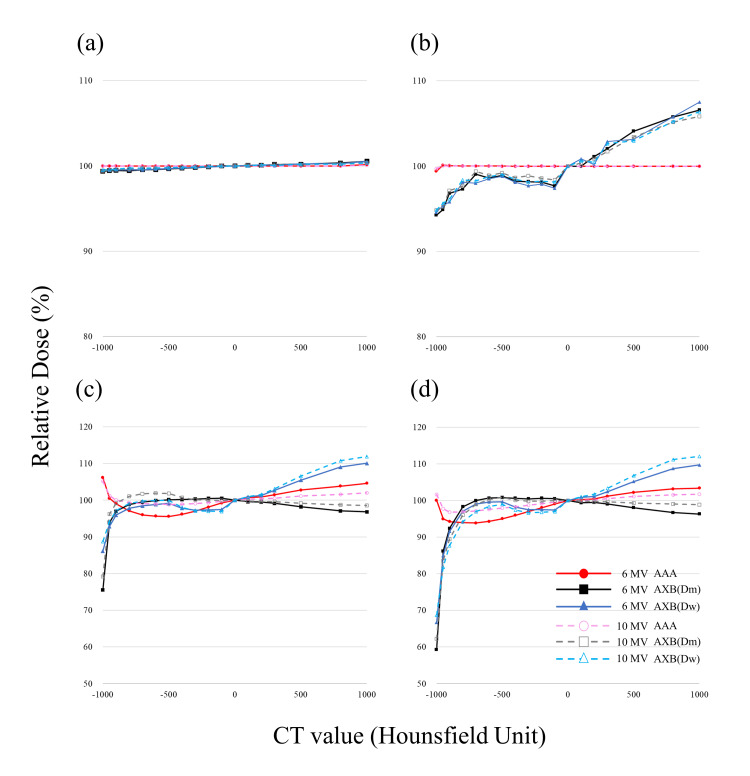
Relative dose for single fields at the center of each VAV as a function of the CT value calculated by each algorithm, normalized with the dose for CT value of 0 HU set to 100%. (A) Central high-density material layer. (B) Central low-density material layer. (C) Central VAV_low_. (D) Central VAV_high_. VAV: variable assignment volume; AAA: analytical anisotropic algorithm; AXB: Acuros XB.

**Figure 6:**
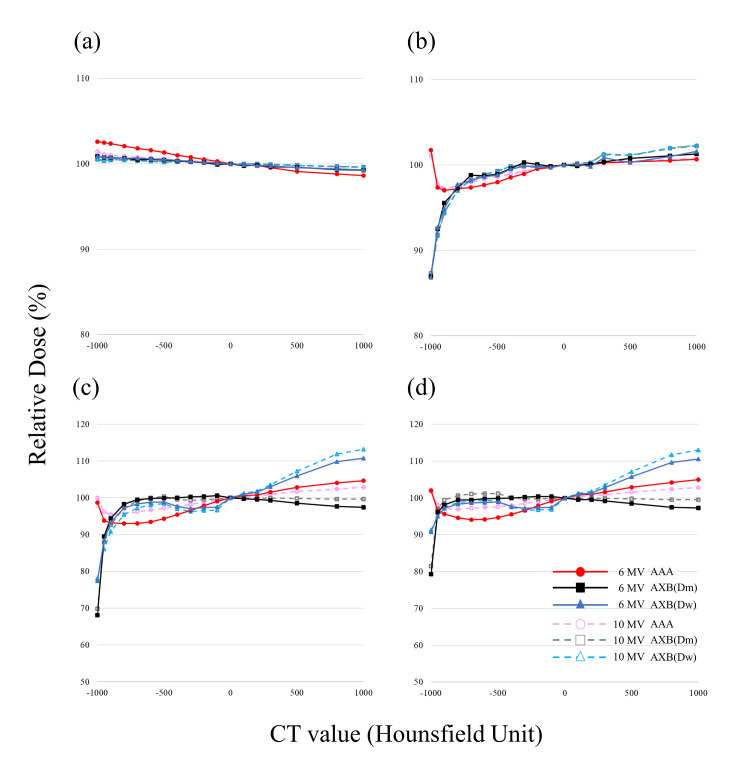
Relative dose for two opposed fields at the center of each VAV as a function of the CT value calculated by each algorithm, normalized with the dose for CT value of 0 HU set to 100%. (A) Central high-density material layer. (B) Central low-density material layer. (C) Central VAV_low_. (D) Central VAV_high_. VAV: variable assignment volume; AAA: analytical anisotropic algorithm; AXB: Acuros XB.

As with the results of the one layer, the RDs for the single and two opposed fields showed similar trends. Figures 5, 6 show the RD profiles for each CT value, with the center of each of the high-density material layer, low-density material layer, VAV_low_, and VAV_high_ as the evaluation points for single fields and two opposed fields (Figures 2B, 2C). The dose at 0 HU was normalized to 100% to evaluate the relationship between CT value and RD for each algorithm. At the center of the high-density material layer, the influence of the CT value of neighboring VAV_low_ was small, and the difference in the RD calculated by AXB with respect to that calculated by AAA at -1000 HU and 6 MV was 0.6% (AXB(m)) and 0.5% (AXB(w)) for the single fields, respectively (Figure 5A). At the center of the low-density material layer, neighboring VAV_high_ showed a deviation from 0 HU at low densities (Figure 5B). At the center of VAV_low,_ differences in the RD for single fields calculated by AXB with respect to that calculated by AAA at 6 MV for -1000 and -800 HU were, respectively, -30.6% and +1.7% (AXB(D_m_)) and -20.0% and -1.0% (AXB(D_w_)). In contrast, at the center of VAV_high_, the differences in the RD calculated by AXB with respect to that calculated by AAA were -40.8% and +4.3% (AXB(D_m_)) and -33.4% and +3.1% (AXB(D_w_)). At the center of VAV_low_ and VAV_high_, the results were similar to that of the one-layer heterogeneous material phantom, regardless of whether the neighboring heterogeneous material was a high-density or low-density material. The RDs calculated by each algorithm and obtained from linear interpolation for CT values of -1000 HU (air), -727 HU (lung), -108 HU (adipose), 26 HU (muscle), 106 HU (cartilage), and 1000 HU (bone) at the centers of VAV_low_ and VAV_high_ are shown in Table 2. The doses shown in the table were examples and the RDs at any CT value could be evaluated.

**Table 2:** Relative doses calculated by each calculation algorithm for CT values for VAVlow and VAVhigh. Relative doses calculated by each calculation algorithm for CT values of -1000 HU (air), -727 HU (lung), -108 HU (adipose), 26 HU (muscle), 106 HU (cartilage), and 1000 HU (bone) for VAV_low_ and VAV_high_, normalized with the dose for CT value of 0 HU set to 100%. VAV: variable assignment volume; AAA: analytical anisotropic algorithm; AXB: Acuros XB.

				Air	Lung	Adipose	Muscle	Cartilage	
				-1000 HU	-727 HU	-108 HU	26 HU	106 HU	1000 HU
Single fields	6 MV	VAV_low_	AAA	106.2%	96.3%	99.1%	100.2%	100.8%	104.6%
			AXB(D_m_)	75.6%	99.3%	100.5%	99.9%	99.6%	96.9%
			AXB(D_w_)	86.2%	98.3%	97.5%	100.2%	100.9%	110.1%
		VAV_high_	AAA	100.1%	93.9%	99.0%	100.1%	100.3%	103.4%
			AXB(D_m_)	59.3%	99.5%	100.5%	99.8%	99.4%	96.3%
			AXB(D_w_)	66.7%	98.5%	97.4%	100.2%	100.9%	109.7%
	10 MV	VAV_low_	AAA	105.2%	99.2%	99.7%	100.1%	100.3%	102.0%
			AXB(D_m_)	79.2%	101.6%	100.0%	99.9%	99.8%	98.6%
			AXB(D_w_)	88.8%	99.6%	96.9%	100.3%	101.1%	111.9%
		VAV_high_	AAA	101.6%	97.1%	99.6%	100.0%	100.1%	101.7%
			AXB(D_m_)	62.3%	98.2%	100.0%	100.0%	99.9%	98.9%
			AXB(D_w_)	68.7%	96.3%	96.9%	100.3%	101.1%	112.1%
Opposed fields	6 MV	VAV_low_	AAA	102.0%	94.2%	99.0%	100.2%	100.8%	105.0%
			AXB(D_m_)	79.3%	99.5%	100.4%	99.9%	99.5%	97.3%
			AXB(D_w_)	90.8%	98.6%	97.5%	100.3%	101.0%	110.6%
		VAV_high_	AAA	98.7%	93.0%	99.0%	100.2%	100.6%	104.7%
			AXB(D_m_)	68.2%	99.2%	100.6%	99.9%	99.8%	97.4%
			AXB(D_w_)	77.4%	98.1%	97.5%	100.3%	101.0%	110.8%
	10 MV	VAV_low_	AAA	101.8%	97.1%	99.5%	100.1%	100.4%	102.8%
			AXB(D_m_)	81.4%	101.0%	99.9%	100.0%	99.9%	99.5%
			AXB(D_w_)	91.3%	99.1%	96.9%	100.3%	101.3%	113.0%
		VAV_high_	AAA	100.0%	96.1%	99.4%	100.1%	100.4%	102.9%
			AXB(D_m_)	69.9%	98.6%	99.7%	100.0%	99.9%	99.7%
			AXB(D_w_)	78.1%	96.7%	96.7%	100.3%	101.3%	113.2%

## Discussion

In this study, we obtained variations in the relative dose for heterogeneous materials calculated by each algorithm with the CT value continuously varied. In the one-layer heterogeneous material phantom, we evaluated the changes in the relative doses calculated by AAA and AXB with the mass density of the heterogeneous material varied using the CT value. The relative dose difference between the two energies was slightly higher in AAA and lower in AXB at lower energy, especially in the high-density region. In AAA, the shorter the path length through the heterogeneous area and the larger the irradiated field, the greater the relative dose difference between the energies. However, the trend of relative dose by CT value was similar in both energies. In the two-layer heterogeneous material phantom, the relative dose at the center of the heterogeneous material was similar to that observed for the one-layer heterogeneous material phantom in the water-equivalent material.

In the air region (CT value of -1000 to -786 HU), the results showed that the dose difference between algorithms AAA and AXB was more than 50%, which indicated the risk of dose reduction or escalation in clinical practice. The longer the path length through the heterogeneous area and the smaller the irradiation field, the larger the dose difference was caused. Rana et al. investigated the accuracy of dose calculations by AXB for water and air beyond the air gap thickness (2, 4, and 6 cm) in simple heterogeneous phantoms [7] by comparing the calculated doses with data measured in different irradiation fields, reporting results similar to that of our study. From this study, it is easy to estimate how much the dose difference between AAA and AXB will be by simply obtaining CT values even in the low-density region where large dose differences are produced.

In the lung region (CT value of -990 to -377 HU), with a photon energy of 6 MV, single irradiation field size of 5 cm × 5 cm, and path length of 5 cm, the relative dose differences for -1000 HU versus -800 HU were -13.1%, +46.0%, and +37.5% for AAA, AXB(D_m_), and AXB(D_w_), respectively. These differences are large, which should be noted when switching between algorithms to determine the dose. On the other hand, Boiset et al. reported that the results of AAA calculations are not significantly different from that of the Monte Carlo calculations for complex stereotactic body radiotherapy (SBRT) cases of lung cancer with heterogeneous tissues and many small irradiated fields, and thus AAA can be used in lung SBRT cases [15]. However, the confluence of AAA and AXB dose calculation results at CT values of -900 to -800 HU (low-density material) in the present study suggests that the CT value plays an important role in the accuracy of dose calculations. Previously, it was only possible to compare doses at specific CT values, but this study allows comparison of doses at any CT value consistent with clinical conditions.

In the high-density region (bone: 106 to 3000 HU), a dose difference of approximately 10% was observed, which was not as large as that in the low-density region. Takizawa et al. reported that AXB(D_m_) provides values that are closer to measured data than AAA, and with large bone volumes, AAA provides overestimates (relative to measured data) in glioma cases with targets including bone (450 HU), air (-1000 HU), and water (-6 HU) [8]. Both Fogliata et al. and Kan et al. reported a discrepancy between AXB(D_m_) and AXB(D_w_) dose calculations for bone using phantoms [5,9]. Similarly, a small reduction in the AXB(D_m_) dose for bone was observed in the present study. With AXB(D_w_), the possibility of hot spots in the bone should be noted [16] because the dose calculated by AXB(D_w_) is generated by converting the dose calculated by AXB(D_m_) with the stopping power ratio for water from the specific medium [17].

It may be possible to compare and evaluate the dose difference dependent on the CT value not only between dose calculation algorithms but also for the same dose calculation algorithm. We found that the relative dose changes with CT value in a present study, and thus the difference in the relative dose may be predicted simply by measuring changes in the CT value, such as density changes from tumor collapse in lung SBRT, adipose fattening from muscle loss during hospitalization, effects of contrast media in contrast studies, and hemorrhage changes. This study was able to complement previous studies that compared AAA and AXB only at specific CT values by visually (graphically) showing that the dose difference between AAA and AXB varies stepwise with CT value and that the direction of the change differs between CT values on the negative and positive side. The study will help to determine in which cases large dose differences occur when shifting from AAA to AXB, and whether these differences are on the positive or negative side.

Furthermore, when assigning CT values for particularly low-density regions, for example, for quality assurance, the results of this study suggest that these regions are where large relative dose changes occur, and thus CT values should be assigned that are consistent with actual conditions.

As a limitation, the present study was a virtual phantom experiment based on the assumptions of a simple system such as single fields or two opposed fields, and further study is needed to determine whether it can be applied to multi-port irradiation, rotational irradiation, and volume prescription in intensity modulated radiation therapy/volumetric modulated arc therapy (IMRT/VMAT) in the clinical setting. In addition, there was no way to perform measurements due to the use of virtual phantoms, because dose to medium cannot be reproduced in the experimental system. According to studies evaluating dose calculations for heterogeneous materials with different densities, AXB(D_m_) provides dose distributions close to those of measurements or Monte Carlo calculations [5-12].

This study focused on the dose difference that occurs when switching between AAA and AXB, the commercial base, and not on identifying the algorithm that performs the correct calculation. Kry et al. compared the differences between D_w,w_, D_m_, and D_w_ to promote consistency in clinical trials by recommending a uniform framework as it relates to radiation transport and dose calculation in water versus in medium [18]. They reported no framework was found to be ideal or perfect given the history, complexity, and current status of radiation therapy. Currently, no calculation algorithm has been found that is consistent with all heterogeneous materials. They also summarized previous studies on soft tissue, bone, and lung, but found no clinical results that contradicted our results. However, since our results indicate that slight differences in CT values (i.e., density) lead to different behavior, our study may be of great significance as a supplement to previous studies.

In the future, based on this study, it may be possible to predict the difference between AAA and AXB dose calculations and the dose difference within each dose calculation algorithm from CT images in the event that only CT values, irradiation fields, and distances through heterogeneous materials are obtained.

## Conclusions

Dose differences between the dose calculation algorithms were clarified for heterogeneous materials with the CT value varied from -1000 to 3000 HU. In particular, in the low-density region (CT value of -1000 to -800 HU), the difference between AAA and AXB doses can be more than 50%, indicating the risk of dose reduction or escalation in the clinical setting. As a result of evaluating the relative dose as a function of continuously varying CT values, changes in the relative dose for each algorithm may be conveniently determined from the change in the physical density.
